# Blinding outcome assessors in randomised controlled trials of psychological interventions: how is it done? A narrative review

**DOI:** 10.1186/s13063-026-09698-0

**Published:** 2026-06-09

**Authors:** Sally O’Keeffe

**Affiliations:** Population Health Sciences Institute, Newcastle University, Newcastle-Upon-Tyne, UK

**Keywords:** Bias, Blinding, Masking, Psychological interventions, Randomised controlled trial

## Abstract

**Background:**

Blinding of patients, clinicians and outcome assessors is a key measure for minimising bias in randomised controlled trials (RCTs). In trials of psychological interventions, where outcome measures often use subjective measures, the blinding of outcome assessors is particularly important. However, there are few guidelines on how blinding of outcome assessors can be achieved in practice. The aim of this study was to identify procedures used for blinding outcome assessors in RCTs of psychological interventions.

**Methods:**

A narrative review was conducted through a PubMed database search covering the period from January 2023 to December 2024, restricted to studies published in the six highest impact factor journals in the field of psychiatry and mental health. Studies were included if they were (1) an RCT and (2) focused on psychological intervention(s). Descriptive statistics were used to summarise the study characteristics, and content analysis of the published reports was conducted to describe the methods used to maintain blinding of outcome assessors.

**Results:**

Fifty-two eligible studies were identified which reported on RCTs of psychological interventions. Of those, the majority reported that outcome assessors were blinded to participants’ treatment allocation (*n* = 44, 85%). Of the studies with blinded outcome assessors, 27 reported information on the procedures used to maintain blinding (61%). Methods for maintaining blinding were avoiding unblinding by participants (*n* = 16), tracking and assessing blinding efficacy (*n* = 12), replacing unblinded assessors (*n* = 11), managing communication within the team (*n* = 9), clear designation of blind and unblind staff roles (*n* = 8) and management of records (*n* = 6). The majority of studies reported a single method for maintaining blinding of outcome assessors.

**Conclusion:**

While the majority of RCTs of psychological interventions reported that outcome assessors were blind to participants’ treatment allocation, there was often no or minimal information about procedures used to maintain blinding. A limitation of this study is that it was restricted to RCTs reported in high-impact journals so it does not provide a comprehensive review of all relevant studies. Maintaining blinding of outcome assessors requires substantial planning and efforts throughout the conduct of an RCT and this should be reported so that conclusions drawn from RCTs can be made with confidence.

**Supplementary Information:**

The online version contains supplementary material available at 10.1186/s13063-026-09698-0.

## Introduction

Randomised controlled trials (RCTs) are considered the gold standard for establishing the evidence for healthcare interventions [1]. This is due to the measures taken to minimise bias to ensure the validity of conclusions drawn from the RCT [1]. Bias is a systematic error in the conduct of a trial that leads to deviation of the measured effect from the true effect of an intervention [2]. One measure to reduce bias in RCTs is blinding, which refers to the process of concealing treatment allocations to the participants, clinicians providing the intervention and research team [3]. Successful blinding in RCTs ensures the validity and credibility of results from RCTs [4, 5].

Failure to blind the research team, particularly those responsible for collecting data—outcome assessors—may result in observer bias. This is where outcomes are influenced by the assessors’ conscious or unconscious bias, which often favours the experimental intervention [6–8]. A study of 228 meta-analyses found exaggerated estimated effects where participants and research teams were not blinded [9]. Studies have found exaggerated treatment effects when outcome assessors were unblinded [6, 7]. This makes blinding a cornerstone of rigorous RCTs.

The Consolidated Standards of Reporting Trials (CONSORT) statement defines a minimum set of items to be included in the reporting of RCTs, and is endorsed globally by hundreds of journals, and has been associated with improvements in the reporting of RCTs [10]. Blinding is listed in the CONSORT statement as an item that should be reported in RCTs [11]. While the importance of blinding in psychological interventions is undisputed, research has found poor reporting of blinding, which compromises critical assessment of the quality of RCTs [12]. A systematic review of RCTs published between 2000 and 2010 of interventions for schizophrenia and affective disorders found blinding was rarely reported or assessed [13]. A more recent study, published in 2021, reviewed RCTs published in high-impact journals and found that blinding was often insufficiently documented [14], indicating that blinding continues to be poorly reported in RCTs. However, these studies have focused on whether blinding was done, rather than *how* it was achieved in practice.

In pharmaceutical research, blinding is relatively straightforward, as the interventions are indistinguishable [5, 8]. Blinding is much more complex in studies of psychological interventions, where there are typically substantial differences between the experimental intervention and control arms. For example, in a study comparing cognitive-behavioural therapy with a waitlist condition, it is not possible to blind the clinicians providing the intervention, nor is it possible to blind the patients receiving it [15, 16]. Another challenge is that such studies often use subjective primary outcomes, such as clinical rating scales that rely on the judgement of an outcome assessor, compared with studies with more objective outcome measures where blinding is less of an issue (e.g. mortality, body weight or blood test results) [17].

Blinding of outcome assessors is therefore critical in psychological interventions, particularly where it is impossible to blind participants and clinicians, and where outcomes may be based on subjective ratings [18]. Some studies have attempted to assess and report on the efficacy of blinding [19]. To do this, outcome assessors are asked to guess the allocation of participants following data collection. However, this practice is controversial. It assumes that outcome assessors’ guesses of treatment allocation should be equivalent to those that would be found by chance if blinding efforts have been successful [20]. However, such assessments cannot distinguish between blindness and hunches about the efficacy of the intervention [21]. For instance, outcome assessors may assume that participants with favourable outcomes received the experimental intervention [22]. On the other hand, outcome assessors may assume that participants whose symptoms have deteriorated are in the control arm.

Blinding is scientifically desirable, yet only 59% of RCTs of psychological interventions published in top psychiatry journals between 2017 and 2018 reported adequate blinding of outcome assessors [14]. Blinding involves significant planning and efforts to maintain in RCTs of psychological interventions [16], yet few studies have reported on how this can be achieved in practice.

A small body of literature advises on methods for blinding of outcome assessors. A commonly reported method for maintaining blinding of outcome assessors is asking participants not to unblind outcome assessors [5, 14, 18, 23]. While participants may be asked not to share their treatment allocation with outcome assessors, they may still do so inadvertently such as by referring to concepts they have learned in therapy [15, 16]. Other procedures for minimising the risk of unblinding outcome assessors include restricting access to study data and databases [8, 18, 23], using independent outcome assessors who are not part of the research team [19], physical separation of outcome assessors and clinicians delivering the intervention [8, 14, 18, 19], or where this is not possible, ensuring vigilance in what is discussed when clinicians and outcome assessors are working in shared spaces [8, 23]. In the event of an outcome assessor being unblinded, subsequent data collection should be conducted by a different outcome assessor [14, 19].

These papers provide useful insight into ways in which blinding can be maintained in RCTs. However, trial teams are increasingly working remotely and across sites, with reliance on electronic communication and record keeping. An updated review on guidelines for blinding procedures was required. This will offer practical guidance for trialists in the planning of future RCTs.

The aim of the present study was to explore methods of blinding outcome assessors in psychological interventions in recent RCTs from high-impact journals. To do this, a narrative review was conducted of psychological trials of blinding of outcome assessors. As there was a dearth of literature on blinding methods of outcome assessors, a narrative review was considered the most appropriate method as it allowed for exploration of the topic to gain further insight.

## Methods

### Searches

A literature search was performed using the PubMed database covering the period from January 2023 to December 2024. The search term ‘trial’ was used to identify clinical trials, and the search was restricted to six journals with the highest impact ratings in the field of psychiatry and mental health, as listed in the Scimago Journal Rank (SJR) website (https://www.scimagojr.com/) in 2025. These journals were *World Psychiatry*, *Lancet Psychiatry*, *Annual Review of Clinical Psychology*, *JAMA Psychiatry*, *Clinical Psychology Review*, and *Psychotherapy and Psychosomatics*. High-impact journals were selected as these are an indicator of methodological quality, to provide insight into contemporary practice in blinding procedures in high-quality RCTs, rather than to provide a comprehensive review of all RCTs published during the study period. The 2 years prior to the search were selected to provide insight into current practice in RCTs.

### Study inclusion and exclusion criteria

Studies were included if they: (1) presented the primary outcome analysis from an RCT and (2) the study focused on a psychological intervention, as judged by the author. Psychological interventions were inclusive of talking therapies, digital interventions and self-help interventions with any intervention with a psychological component. Studies were excluded if they were not an RCT or if they were secondary analyses of RCTs. Titles and abstracts were screened to assess eligibility.

### Data extraction strategy

For all included studies, the full text was read (including supplementary materials) and the following data extracted and inputted into an Excel spreadsheet: (1) author; (2) primary outcome(s); (3) primary outcome type(s) (Assessor-rated; Self-report/Objective); (4) who collects data; (5) blind outcome assessors (Yes/No/N/A/Unspecified); and (6) blinding methods reported (Yes/No/N/A/Unspecified) (see Table 1). Detail on how blinding was maintained was extracted and compiled in NVivo V14.0 [76].

**Table 1:** Details of the eligible studies

Authors	Primary outcome(s)	Primary outcome type(s)	Who collects data	Blind outcome assessors	Blinding methods reported
Assmann et al. [24]	Borderline Personality Disorder Severity Index	Assessor-rated	Outcome assessors	Yes	No
Babiy et al. [25]	Edinburgh Postnatal Depression Scale	Self-report	Online	N/A	N/A
Binder et al. [26]	Perceived Stress Scale	Self-report	Independent clinicians	Yes	No
Blom et al. [27]	Insomnia Severity Index; Montgomery-Åsberg Depression Rating Scale	Self-report	Outcome assessors	Yes	Yes
Boudreaux et al. [28]	Death by suicide or suicide-related acute health care visits (from medical records)	Objective	Unspecified	Unspecified	Unspecified
Brown et al. [29]	Mood and Feelings Questionnaire	Self-report	Outcome assessors	Yes	Yes
Bryant et al. [30]	Clinician-Administered PTSD scale 2	Assessor-rated	Independent clinicians	Yes	Yes
Bryant et al. [31]	Prolonged Grief-13 Scale	Self-report	Independent clinicians	Yes	Yes
Castro-Camacho et al. [32]	Patient Health Questionnaire-9; PTSD checklist for DSM-5	Self-report	Outcome assessors	Yes	No
Cooperman et al. [33]	Days until first illicit drug use through ecological momentary assessment or positive drug screen	Objective and self-report	Outcome assessors	Yes	Yes
Creswell et al. [34]	Child Anxiety Impact Scale–parent report	Self-report	Outcome assessors	No	N/A
Dafsari et al. [35]	Geriatric Depression Scale	Self-report	Outcome assessors	Yes	Yes
Daumit et al. [36]	Tobacco abstinence (biochemically validated)	Objective	Outcome assessors	Yes	Yes
Diefenbach et al. [37]	Columbia Suicide Severity Rating Scale	Assessor-rated	Outcome assessors	Yes	Yes
Ehlers et al. [38]	PTSD Checklist for DSM-5	Self-report	Outcome assessors	Yes	Yes
Fink et al. [39]	Self-Reporting Questionnaire	Self-report	Outcome assessors	Yes	No
Freeman et al. [40]	Psychotic Symptoms Rating Scale–Delusions	Assessor-rated	Outcome assessors	Yes	Yes
Gardner et al. [41]	Use of benzodiazepine receptor agonist (BZRA), assessed by self-report and through no dispensing of a BZRA	Objective and self-report	Outcome assessors	No	N/A
Goldstein et al. [42]	Columbia Suicide Severity Rating Scale; ALIFE Self-Injurious/Suicidal Behavior Scale; ALIFE Psychiatric Status Rating	Assessor-rated	Outcome assessor	Yes	Yes
Goulding et al. [43]	Time to relapse (weeks to new mood episode based on ratings using a Longitudinal Interval Follow-up Evaluation Clinical Monitoring Form)	Assessor-rated	Outcome assessors	Yes	Yes
Hankin et al. [44]	Symptom Checklist-20; Edinburgh Postnatal Depression Scale	Self-report	Outcome assessors	Yes	No
Hautzinger et al. (2024) [45]	Longitudinal Interval Follow-Up Evaluation Psychiatric Status Rating	Assessor-rated	Independent clinicians	Yes	Yes
Hertenstein et al. [46]	Insomnia Severity Index; Glasgow Sleep Impact Index	Self-report	Unspecified	Unspecified	Unspecified
Hoge et al. [47]	Clinical Global Impression of Severity scale	Assessor-rated	Outcome assessors	Yes	No
Janssen et al. [48]	Quick Inventory of Depressive Symptomatology—Self Report	Self-report	Outcome assessors	Yes	No
Juul et al. [49]	Zanarini Rating Scale for Borderline Personality Disorder	Assessor-rated	Outcome assessors	Yes	Yes
Karatzias et al. [50]	International Trauma Questionnaire	Self-report	Outcome assessors	Yes	No
Kopf-Beck et al. [51]	Beck Depression Inventory-II	Self-report	Outcome assessors	Yes	Yes
Lincoln et al. [52]	Psychotic Symptoms Rating Scales	Assessor-rated	Outcome assessors	Yes	Yes
Liu et al. [53]	PsychoSocial Index	Self-report	Independent clinicians	Yes	Unspecified
Löwe et al. [54]	Patient Health Questionnaire-9	Self-report	Outcome assessors	Yes	Yes
Maulik et al. [55]	Patient Health Questionnaire-9; Mental Health Knowledge, Attitude and Behavior scale	Self-report	Outcome assessors	Yes	No
McGorry et al. [56]	Global Functioning: Social and Role scales	Assessor-rated	Outcome assessors	Yes	Yes
Meganck et al. [57]	Hamilton Rating Scale for Depression	Assessor-rated	Outcome assessors	Yes	No
Miller et al. [58]	Insomnia Severity Index; percent heavy-drinking days; alcohol problems from 13-item version of the Short Inventory of Problems	Self-report	Outcome assessors	Yes	No
Mueller-Weinitschke et al. [59]	Quick Inventory of Depressive Symptomatology	Assessor-rated	Outcome assessors	Yes	No
Reininghaus et al. [60]	Rosenberg Self-Esteem Scale	Self-report	Outcome assessors	Yes	Yes
Schaich et al. [61]	Hamilton Rating Scale for Depression	Assessor-rated	Outcome assessors	Yes	No
Schramm et al. [62]	Hamilton Rating Scale for Depression	Assessor-rated	Outcome assessors	Yes	Yes
Seinsche et al. [63]	Heart rate/heart rate variability; positive and negative feelings	Objective and self-report	Unspecified	Unspecified	Unspecified
Sharifi et al. [64]	Strengths and Difficulties Questionnaire—parent report	Self-report	Outcome assessors	Yes	No
Sharpe et al. [65]	Days spent as an inpatient (from medical records)	Objective	Outcome assessors	Yes	No
Slade et al. [66]	Manchester Short Assessment	Self-report	Online	N/A	N/A
Sloan et al. [67]	Clinician-Administered PTSD Scale for DSM-5	Assessor-rated	Outcome assessors	Yes	Yes
Strauss et al. [68]	Patient Health Questionnaire-9	Self-report	Outcome assessors	Yes	Yes
Waite et al. [69]	Insomnia Severity Index	Self-report	Outcome assessors	Yes	Yes
Walburg et al. [70]	Body weight change	Objective	Clinical team	No	N/A
Watkins et al. [71]	Patient Health Questionnaire-9	Self-report	Outcome assessors	Yes	Yes
Wolf et al. [72]	Yale-Brown Obsessive-Compulsive Scale	Assessor-rated	Outcome assessors	Yes	Yes
Wong et al. [73]	Sky Search subtest of the Test of Everyday Attention for Children	Objective	Outcome assessors	Yes	No
Zarski et al. [74]	Hamilton Anxiety Rating Scale; Quick Inventory of Depressive Symptomatology	Assessor-rated	Independent clinicians	Yes	Yes
Zuccolo et al. [75]	Edinburgh Postnatal Depression Scale	Self-report	Independent clinicians	Yes	Yes

*DSM-5 *Diagnostic and Statistical Manual of Mental Disorders—5th edition, *PTSD *post traumatic stress disorder

### Data synthesis and presentation

Descriptive statistics were used to summarise the proportion of trials that had blinded outcome assessors, and of those, how many reported blinding procedures. Conventional content analysis was used [77] to synthesise procedures used to maintain blinding. To do this, all information relevant to the blinding of outcome assessors was extracted from the full-text papers and supplementary materials and stored in NVivo. An inductive approach was used. Codes were applied to all information in NVivo, which were grouped into categories and subcategories based on their similarities and differences. These are reported in the order of frequency with which they were reported.

## Results

**Fig. 1:**
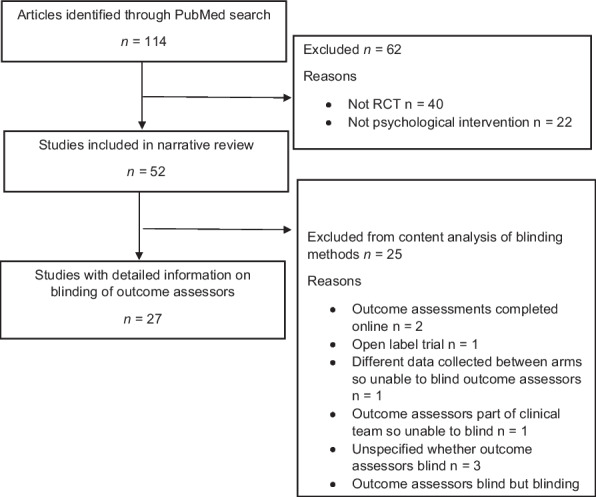
Flow diagram for identification of eligible trials

One hundred fourteen potentially relevant articles were identified using the search strategy described above and the titles and abstracts were screened. Sixty-two were excluded as they did not meet eligibility criteria, as they were not RCTs (*n* = 40) or they were not studies of psychological interventions (*n* = 22). Fifty-two were retained for full-text search and of these, all met the inclusion criteria and were included in the narrative review (see Fig. 1). Details of the eligible studies are shown in Table 1.

### Blinding of outcome assessors

Of the 52 eligible studies, the majority reported that outcome assessors were blind to participants’ treatment allocation (*n* = 44, 85%). Of the remaining eight studies, two collected primary outcome data online which meant blinded outcome assessors were not needed [25, 66]. Three studies reported that outcome assessors were unblinded (reasons were open-label trial [41], differences in the data collected between arms [34] and outcome assessors part of the clinical team [70]). Three studies did not specify whether outcome assessors were blinded [28, 46, 63].

Table 1 summarises the study characteristics for all 52 included studies. This shows that different types of outcome measures were used across the 52 studies: the majority relied upon self-report questionnaires (*n* = 26), followed by assessor/clinician-rated measures (*n* = 18), and a minority relied upon more objective measures (*n* = 5) or a combination of objective and self-report measures (*n* = 3). Assessor-rated measures are arguably the most susceptible to assessor bias due to the subjectivity in rating such measures. It is encouraging to note that all 18 studies using assessor-rated measures did report that outcome assessors were blinded, although five did not state the methods used to maintain blinding of outcome assessors.

### Procedures for blinding outcome assessors

Of the 44 studies that reported that outcome assessors were blind to the allocation of participants, 27 reported information on the procedures used to maintain blinding (61%). Content analysis of these 27 studies found 28 methods of procedures for managing blinding of outcome assessors, which were grouped into 6 categories. The six categories are described below.

#### Managing risk of unblinding by participants (*n* = 16)

A commonly reported approach to avoiding unblinding of outcome assessors was to minimise the risk of unblinding by participants. This often involved informing participants not to notify outcome assessors about their allocation and reminding them of this at each research assessment [29, 35, 36, 38, 45, 49, 52, 62, 67, 69, 71, 72]. Some studies further specified that participants were asked not to comment on specific aspects of the interventions that may unblind the outcome assessors [62, 75]. One study had a separate unblinded researcher who collected data on one measure which would have unblinded the outcome assessors [43]. One study collected outcome data online where possible to minimise the risk of bias by limiting communication with the research team, thus reducing opportunities for participants to disclose treatment-related information [68].

#### Tracking unblinding and assessing blinding efficacy (*n* = 12)

Studies frequently reported that unblinding was tracked. This was done through outcome assessors recording when they were unblinded [36, 40, 56, 60, 69], through them guessing the allocation of participants [30, 31, 35, 71] or both [27, 37, 52]. Guesses were sometimes reported to estimate the efficacy of blinding [30, 31].

#### Managing incidences of unblinding: replacing outcome assessors (*n* = 11)

The next most commonly reported method was approaches to re-establish blinding when outcome assessors had become unblinded. When unblinding occurred, it was often reported that a different outcome assessor who was blinded collected subsequent data [40, 51, 52, 60, 67–69, 72, 74, 75]. Two studies reported that assessments were recorded, which enabled them to be re-rated by a blinded rater in the event of the outcome assessor being unblinded [38, 52]. To do this, the recording was masked to remove any information that would potentially unblind the new rater.

#### Managing communication within the team (*n* = 9)

Studies often reported ways in which communication within the team was managed to avoid the risk of unblinding outcome assessors. Most commonly, studies reported locating outcome assessors and clinicians at different locations [35, 40, 42, 52, 62, 67, 69] and asked clinicians to refrain from discussing details of individual treatments with outcome assessors [35, 52, 62, 72]. Other approaches included holding training to ensure the team understood the principles of blinding [52], asking team members to be cautious when making phone calls in shared office space [52], shielding outcome assessors from treatment-related discussions [52, 75], reminding team members about blinding at each team meeting [42] and asking outcome assessors to leave team meetings prior to treatment-related discussions [42].

#### Clear designation of blind and unblind staff roles (*n* = 8)

A number of studies reported managing blinding through clear designation of which staff roles were blinded and unblinded. Three studies reported that an unblinded team member notified participants of their randomisation outcome [33, 68, 69]. Other approaches included ensuring that clinicians were not involved in outcome assessments [52] and that outcome assessors were not involved in intervention delivery [36] or recruitment [54]. Some studies reported the use of independent fidelity raters [30] and outcome assessors [74], who were not otherwise involved in the study.

#### Management of records (*n* = 6)

A minority of studies reported ways in which records were managed to minimise the risk of unblinding of outcome assessors. This included not providing outcome assessors with access to electronic records that would unblind them or having separate databases from unblinded team members [42, 52, 71], not providing access to clinical notes to outcome assessors [40, 69] and storing documents at a separate physical location from outcome assessors so they could not access unblinding information [35].

### Number of blinding methods reported per study

**Fig. 2:**
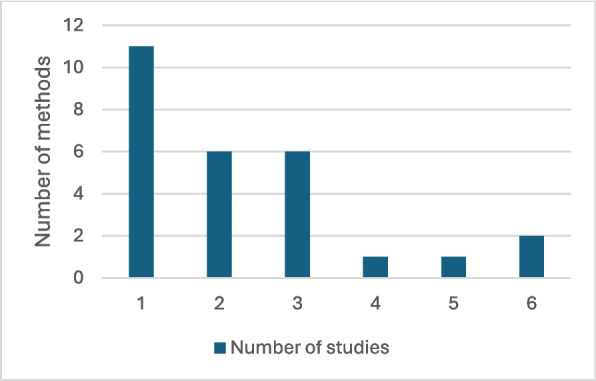
Number of methods for maintaining blinding of outcome assessors reported

Figure 2 shows the number of methods of blinding reported per study, based on the six categories of blinding methods identified in the content analysis. This shows that the majority of studies reported just one method of blinding (*n* = 11, 41%). Six studies reported two methods (22%) and six studies reported three methods (22%). Few studies reported four or more methods, and only two studies reported using all six blinding methods identified in the content analysis [52, 69].

## Discussion

Of the 52 eligible studies that reported on RCTs of psychological interventions in high-impact journals between 2023 and 2024, the majority reported that outcome assessors were blind to participants’ treatment allocation (*n* = 44, 85%). This shows an upward trend in recent years, as an earlier study found 47% of trials in leading general medicine journals published between 2002 and 2003 reported blinding of outcome assessors [78]. In the present review, where outcome assessors were not blinded, this was typically justified (e.g. due to outcome assessments being completed online or due to practical issues making it unfeasible to blind outcome assessors). However, three studies did not specify whether outcome assessors were blind to treatment allocation. Similar to the earlier review by Juul and colleagues [14], a substantial proportion (39%) of studies in the present study did not provide sufficient detail on how blinding was managed in practice. Given exaggerated treatment effects when outcome assessors are unblinded [6, 7], it is crucial that sufficient detail on blinding methods is reported in RCTs so that conclusions drawn from these studies can be made with confidence [14]. CONSORT states that blinding status should be reported for outcome assessors, and how blinding was achieved [10]. Thus, many studies are falling short of this standard and improved reporting is required.

Of the 44 studies with blinded outcome assessors, 27 reported information on the procedures used to maintain blinding (61%). Content analysis of these studies identified a range of methods for maintaining blinding. Table 2 summarises these to provide a list of considerations for trialists when planning methods for maintaining blinding of outcome assessors. The most common method reported was avoiding unblinding by participants through asking them not to discuss their treatment, a method frequently reported in the existing literature [5, 14, 18, 23]. Previous studies have frequently reported replacing outcome assessors in the event they become unblinded [14, 19], an approach that was frequently reported in studies in the present review.

**Table 2:** Considerations for potential sources of unblinding and mitigations

Potential source of unblinding	Specific ways in which unblinding may occur	Mitigations
Data collection and analysis	Participant mentions treatment-related information	• Explain to participant the need for outcome assessor to be blinded and remind them at each interaction not to discuss treatment-related information
	Participant discloses treatment allocation during clinician-rated assessment tool	• Assessment is audio/video-recorded. Treatment information can be masked on the recording and then re-rated by a blinded rater
	Different measures used between treatment arms, e.g. therapeutic alliance questionnaire in the experimental arm	• Measures that would unblind outcome assessors either: ○ Administered by separate unblinded assessor; or ○ Administered by online questionnaire or text message
	Measures that may illicit information about treatment, e.g. service use data for economic evaluation	• Measures that would unblind outcome assessors administered by separate unblinded assessor, or collected from medical records (where possible)
	Conducting qualitative interviews which explore experiences of the intervention	• Interview facilitated by unblinded researcher • If outcome assessor is required to conduct qualitative interviews, this should be done after outcome data collection is complete
	Qualitative transcription and analysis of interviews for process evaluation	• Transcription of interviews and qualitative analysis to be conducted by unblinded team members • If transcription and analysis is to be conducted by the outcome assessors, this should be carried out after the outcome data collection is complete
	Treatment fidelity assessments which may involve listening to recordings of intervention sessions or extracting information from medical records	• Fidelity data to be collected by unblinded member of the team or an independent rater • If fidelity assessments are to be carried out by outcome assessors, this should be done after outcome data collection is complete
Study records	Through access to study databases holding treatment information (e.g. treatment allocations, treatment adherence information, adverse events logs, recordings of intervention sessions, health use data, qualitative interview data, treatment fidelity data)	• Electronic files organised in a way that separates information required by blinded (e.g. patient contact information) and unblinded staff (e.g. treatment information) • Access to treatment information restricted to unblinded staff only through password-protected files
	Through access to paper records of trial documentation which hold treatment information	• Paper records with treatment-related information to be stored in a locked filing cabinet that outcome assessors do not have access to. Separate filing cabinets should be used by blinded and unblinded staff
Communication	Outcome assessor included in discussions about treatment information, or overhears information by accident	• Outcome assessors and clinicians located at different locations • Clinicians asked to refrain from discussing details of individual treatments with outcome assessors • Training and reminding staff regularly about the importance of blinding • Reminding unblinded staff to be aware when discussing treatment information or making phone calls about treatment in shared offices • Having a section of team meetings for discussion of treatment-related issues, where blinded outcome assessors are asked to leave the meeting
	Treatment information needing to be communicated (e.g. notifying randomisation outcomes to participants and clinical team)	• Communication regarding treatment to be undertaken by staff designated an unblinded
	Outcome assessor unblinded by having duties relating to treatment	• Outcome assessors not involved in delivery of intervention • Outcome assessors not involved in recruitment so their presence at the trial sites is not required • Outcome assessors independent of all other trial-related activity (e.g. qualitative interviews and fidelity assessments) • Not involving clinicians in outcome assessments

Another commonly reported method was tracking and assessing blinding efficacy. Several studies tested the efficacy of blinding through asking outcome assessors to guess the allocation of participants [27, 30, 31, 35, 37, 52, 71]. This is despite this practice being considered problematic, as such assessments cannot distinguish between blinding efficacy and outcome assessors’ hunches about the efficacy and harms of the intervention [14, 21]. It may be preferable to base blinding efficacy estimates on outcome assessors’ reports of whether they have been unblinded, rather than their guesses, as was done in a number of studies [36, 40, 56, 60, 69].

A key blinding method reported was managing communication within the team which incorporated a range of approaches to maintain blinding. These efforts included locating outcome assessors and clinicians at different locations, training and frequent reminders about blinding within the team and asking trial staff to be mindful of who was present when discussing treatment-related information (e.g. when making phone calls in shared spaces and asking outcome assessors to leave team meetings during treatment-related discussions). These approaches overlap with those reported in previous studies [8, 14, 18, 19, 23] and reflect the many considerations required to ensure blinding of outcome assessors.

Another key blinding method reported was clear designation of staff roles between those who were blinded and unblinded. In these studies, the teams ensured that tasks were assigned according to blinding status, such as having an unblinded team member notify participants of their randomisation outcome [33, 68, 69], clinicians not conducting outcome assessments [52], and outcome assessors not being involved in intervention delivery [36] or recruitment [54]. Some studies reported the use of independent fidelity raters [30] and outcome assessors [74], who were not otherwise involved in the study. The use of independent outcome assessors is perhaps the ideal scenario to minimise the risk of unblinding, yet in reality, this is not always practical. Many studies will be conducted by a core research team, where outcome assessors are responsible for the day-to-day running of the trial, so their duties cannot be restricted to just outcome assessments. In studies where it is not possible to use independent outcome assessors, it is important to review all potential avenues for unblinding to mitigate these. For example, fidelity assessments are a key component of RCTs to ensure that psychological interventions are delivered as intended. Intervention fidelity assessments may involve listening to recordings of intervention sessions or extracting information from medical records. This is often resource-intensive work that cannot be undertaken by blinded staff. Similarly, qualitative process evaluations are now commonplace within RCTs of psychological interventions [79], typically involving in-depth interviews with trial participants which are then transcribed. Conducting qualitative interviews and their transcription and analysis are resource-intensive tasks and require unblinded staff to undertake these duties, so it is crucial to ensure that RCTs are resourced sufficiently so that these activities can be conducted without compromising the blinding of outcome assessors.

Consideration must be paid to data collection measures that could unblind outcome assessors. For instance, if measures are used that are only applicable to the experimental arm [34] or measures that ask about treatment, such as measures of healthcare use for economic evaluations [80]. Approaches to avoiding unblinding in such cases may include the use of online data collection [66] or separate assessors collecting data that poses risk of unblinding [43]. Again, these issues are crucial to consider at the outset of planning an RCT. The use of online data collection is a potential innovation that may counteract blinding issues, as it ensures that measures can be presented in exactly the same way across study arms. It is, however, important to consider the potential impact on retention rates, which often presents a significant challenge in RCTs [81]. A neglected area from studies included in this review was the management of adverse events. Outcome assessors may become aware of adverse events or risk or safeguarding issues which need to be reported, which may require liaison with clinical teams. These tasks require clear designation of roles to ensure blinding is maintained and how it will be managed in the event the outcome assessor is unblinded. This may prove more difficult in small teams where resources are limited. This is a key consideration in ensuring that RCTs are sufficiently resourced.

A blinding method reported in a minority of studies was consideration of the management of trial records and databases. In the past, trial documentation would have typically been in paper format which may have been easier to separate unblinded information with locked filing cabinets that outcome assessors did not have access to. The conduct of RCTs has changed with the increase in remote working, with study documentation such as the trial master file and investigator site files now often being electronic [82]. These hold potentially unblinding information, such as randomisation forms. It is critical that consideration is given to how study documents and databases are managed to ensure access to allocation lists is restricted to unblinded staff only. This may be achieved through restricted access or passwords held only by unblinded staff, while blinded staff may have separate databases (e.g. with patient contact information).

While 61% of studies included in this review reported some information on blinding methods, studies typically reported use of a single method to maintain blinding (e.g. reminding participants not to discuss details of their treatment with the outcome assessor). In reality, blinding of outcome assessors in complex RCTs of psychological interventions is likely to involve numerous approaches embedded across all aspects of the trial conduct. An excellent example by Lincoln and colleagues provided an in-depth description which spanned across all six categories of blinding methods identified in the content analysis [52]. This included maintaining separation of outcome assessors from unblinded staff through separate electronic databases and office space where possible, to avoid outcome assessors overhearing conversations about individual treatments. They reported that education of blinding was continuous throughout the project, and when unblinding did occur, it was managed through recording it and replacing the outcome assessors for subsequent data collection [52]. Another excellent example by Waite et al. described specific details such as considerations around room bookings [69]. For instance, in a trial comparing the experimental intervention with a waitlist condition, where outcome assessors and clinical staff work in the same building, an outcome assessor seeing a participant arriving for their intervention session would inadvertently unblind them. These provide examples of how maintenance of blinding requires consideration of minute details of the day-to-day running of RCTs, and such methods will be interwoven into all aspects of the running of the trial, such as desk arrangements and consideration to where intervention sessions and phone calls may take place. Blinding procedures are not always reported even when procedures have been adequately implemented [83, 84]. Studies that provided extensive detail about all blinding methods strengthen confidence in the validity of findings [52, 69]. This practice is to be encouraged and will help establish best practice guidelines in blinding methods.

This study had limitations which are important to note. Firstly, this narrative review sought to provide insight into the methods used for blinding outcome assessors. While a systematic approach was used to search studies published in high-impact journals over the study period, this review did not seek to assess the quality of the published reports. The review focused on reports in high-impact journals, so it is unknown how representative these findings are of RCTs published in lower ranking journals. Finally, the review only examined RCT reports (including the main RCT report and supplementary materials). It is possible other blinding methods may have been used in these RCTs that may have been reported elsewhere (e.g. in the trial protocol) or that could have been obtained through contacting corresponding authors.

### Future directions for research

Further work is required to continue to improve blinding methods. Table 2 summarises the efforts that can be made to maximise blinding of outcome assessors based on what we have learnt from this review. However, there may be a range of innovative methods used to ensure the blinding of outcome assessors that are being used in practice. Qualitative studies with trial staff would be beneficial to fully explore the range of approaches used to ensure blinding is maintained to contribute to best practice guidelines. Qualitative studies with patients would also be valuable to explore how blinding can be communicated with them most effectively to reduce the risk of unblinding outcome assessors. Future empirical studies should compare which methods are most successful at preventing unblinding. The development of objective metrics for assessing and reporting the effectiveness of blinding procedures in future trials would represent an important step toward improved transparency.

## Conclusion

This study found that while the majority of RCTs of psychological interventions published in high-impact psychiatry and mental health journals between 2023 and 2024 reported that outcome assessors were blind to participants’ treatment allocation, yet there was typically minimal information provided on the procedures used to maintain blinding. Adequate reporting of blinding methods is necessary so that valid conclusions can be drawn from RCTs. Adequate reporting will inform best practice in blinding procedures. Maintaining blinding of outcome assessors requires substantial planning and efforts throughout the conduct of an RCT, with considerations spanning across communication between participants, clinicians and research teams to organisation of trial records. This has implications for funding of RCTs, as trial teams must be sufficiently resourced to enable outcome assessors to remain blinded.

## Supplementary Information

Supplementary Material 1.

## Data Availability

All data generated or analysed during this study are included in this published article and its supplementary information files.

## Abbreviation

RCT: Randomised controlled trial
